# Physicochemical and physiological changes during the ripening of Banana (*Musaceae)* fruit grown in Colombia

**DOI:** 10.1111/ijfs.14851

**Published:** 2020-11-03

**Authors:** Jhon Larry Moreno, Thierry Tran, Borja Cantero‐Tubilla, Karina López‐López, Luis Augusto Becerra Lopez Lavalle, Dominique Dufour

**Affiliations:** ^1^ The Alliance of Bioversity International and the International Center for Tropical Agriculture (CIAT) CGIAR Research Program on Roots Tubers and Bananas (RTB) Apartado Aéreo 6713 Cali Colombia; ^2^ Facultad de Ingeniería y Administración Universidad Nacional de Colombia (UNAL) Carrera 32 # 12‐00 Palmira Valle del Cauca Colombia; ^3^ Qualisud University of Montpellier, CIRAD, SupAgro, University of Avignon, University of La Réunion 73 rue JF Breton Montpellier 34398 France; ^4^ CIRAD UMR Qualisud Montpellier F‐34398 France; ^5^ Robert Frederick Smith School of Chemical and Biomolecular Engineering Cornell University Ithaca NY 14850 USA; ^6^ Facultad de Ciencias Agropecuarias Universidad Nacional de Colombia (UNAL) Carrera 32 # 12‐00 Palmira Valle del Cauca Colombia

**Keywords:** Climacteric fruit, colour, peel, plantain, respiration, ripening, soluble solids, starch

## Abstract

The physicochemical and physiological attributes of three contrasting commercial varieties of *Musaceae,* Dominico Harton (plantain), Guineo (cooking banana) and Gros Michel (dessert banana), were evaluated and statistically analysed during post‐harvest ripening. Quality attributes differed markedly among varieties, both in fresh fruits and during ripening. Variety (V) had a significant effect (*P* < 0.001) on all attributes except total soluble solids (TSS), carotenes and total chlorophyll. Storage time (ST) had a significant effect on all attributes except colour parameter b* and total carotenes. Starch levels decreased significantly (*P* < 0.001) during ripening, with nearly complete hydrolysis in Gros Michel, followed by Guineo and Dominico Harton. Discriminant analysis showed that central diameter, TSS of the pulp, colour parameter a* and total starch had the highest weight in the differentiation among varieties. These results point out which parameters may help improve current methods for monitoring ripening of bananas, in particular the commercially important varieties in this study.

## Graphical abstract

Three contrasted banana varieties exhibited different behaviors during ripening. Comprehensive assessment and correlation analysis of quality parameters (color, peel and pulp physicochemical characteristics, respiration) identified two non‐destructive parameters, central diameter and color parameter a*, that can predict quality indicators of the ripening stage such as TSS and total starch of the pulp.

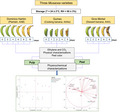

## Introduction

Bananas (*Musaceae* AAA, AA) and plantains (*Musaceae* AAB, ABB) are one of the world's main food resources and play an important socio‐economic role in developing countries in tropical and sub‐tropical regions. The world production for bananas and plantains in 2017 was 113 and 39 million tons, respectively (FAO (Food & Agriculture Organization of the United Nations), 2019). In Colombia, varieties from the Cavendish subgroup are grown mainly for the export trade, thanks to their characteristics such as slow ripening and tolerance to impacts during handling. Other banana varieties are produced for the national market; for instance, Gros Michel remains popular although it is no longer produced for export following the Fusarium wilt R1 outbreak in the 1960s (Ploetz, 2015; Dita *et al*., 2018). Plantain varieties are cultivated traditionally as part of the subsistence economy by small producers, with high geographical dispersion and generation of rural employment. Colombia is among the five main producers in the world, with a production of more than 3.5 million tons of plantain (FAO (Food & Agriculture Organization of the United Nations), 2019), of which 3.3% are exported, 1% is used by the agro‐industry, 10% are post‐harvest losses, and the rest is consumed in rural and urban households (Minagricultura (Ministerio de Agricultura de Colombia), 2019).

Bananas and plantains are climacteric fruits. Their ripening is the result of transcriptional regulation (Yan *et al*., 2019), associated with an increase in the respiration rate and autocatalytic synthesis of ethylene (Johnson *et al*., 1997; Gamage & Rehman, 1999). The action of ethylene results in softening of the fruit, acceleration of deterioration and shortening of post‐harvest shelf‐life (Saltveit, 1999). The climacteric peak triggers various physiological and physicochemical changes: conversion of starch to sugars (Hill & Ress, 1995), enzymatic degradation of the structural carbohydrates (Kojima *et al*., 1994) and degradation of the chlorophyll (Thomas & Janave, 1992). These changes affect the organoleptic attributes of the fruit as well as commercial value, and need to be controlled to minimise losses (Liu *et al*., 2013). Gibert *et al*. (2009) highlighted a strong relationship between varietal characteristics and consumption patterns, in particular for the plantain subgroup in relation to users' preferences including producers, processors and consumers. Consumers evaluate the quality of the fruit mainly by colour, brightness and size. These criteria are complemented by texture (firmness), total soluble solids (TSS) and acidity, in the case of bulk buyers with access to quality control facilities.

In commercial practice, the ripening of banana fruits is controlled by modifying the environment, including temperature (Yang *et al*., 2011; Zhu *et al*., 2018), relative humidity, ethylene concentration and ethylene inhibitors (Jiang *et al*., 1999). Optimum storage conditions typically use temperatures above 15 °C and relative humidity of 92%. Nevertheless, shelf‐life also depends on the variety, independently from post‐harvest environmental conditions (Nunes *et al*., 2013). Several studies have investigated post‐harvest handling of bananas and plantains with the aim of reducing losses (Lo'ay & Dawood, 2017; Alali *et al*., 2018; Liu *et al*., 2019; Lo'ay & EL‐Khateeb, 2019; Khademi *et al*., 2019; Al‐Qurashi & Awad, 2019). Other studies have focused on the acceptability, in terms of shelf‐life and post‐harvest quality (peel and pulp weight, soluble solids, sugars, starch), of bananas from genotypes screened for their tolerance to diseases, such as black Sigatoka (Oiram Filho *et al*., 2019) and Fusarium wilt R1 and TR4 (Smith *et al*., 2014). Fusarium wilt TR4 is a major concern in Colombia with the outbreak reported in the north of the country in August 2019. To our knowledge, no studies have been published on the effect of TR4 on post‐harvest quality, as affected plants wither quickly without producing fruits.

Few studies have been reported on the ripening of Colombian varieties of bananas and plantains, from harvest to post‐harvest storage and subsequent senescence. This work characterises the physicochemical and physiological attributes of three contrasting commercial varieties of *Musaceae* (Dominico Harton, Guineo and Gros Michel) during post‐harvest storage, with the objectives of identifying (i) which attributes best discriminate among varieties using multivariate statistical techniques, and (ii) which attributes may be most suitable as predictors of maturity and quality of ripening bananas and plantains.

## Materials and methods

### Materials

Three commercial *Musa* varieties were evaluated, including dessert banana (Gros Michel, AAA), plantain (Dominico Harton, AAB) and cooking banana (Guineo, AAA). The fruits were harvested from Armenia, department of Quindío, Colombia (1360 m above sea level), purchased and delivered to the Post‐harvest Quality laboratory at CIAT on the same day, which was recorded as Day 0. On the day of harvest, the fruits were in the entirely green state of ripeness (Wang *et al*., 2014), between 8 and 10 weeks after flowering. Storage under controlled conditions began immediately upon delivery.

### Ripening kinetics

In a first experiment, hands of the three *Musaceae* varieties (i.e. clusters of 5 to 8 fruits close to each other on the banana bunch) were stored at 24 °C and 60% relative humidity and their ripening times were evaluated. The hands were placed with the inside curvature facing up, and the individual fruits (fingers) were numbered from left to right. The fruits were stored in a normal day/night cycle. The colour of the peel at the inner and outer curve of the fruit at mid‐length, and the total soluble solids (TSS) of the pulp were measured for selected fruits at various ripening time points. These time points ranged from the day of harvest to the fruit senescence, identified visually as the day when the fruits reached a stage of full‐ripeness (peel colour entirely yellow with brown spots, Wang *et al*, 2014). Senescence depended on the variety: For Dominico Harton, 144 fruits distributed among 21 hands were evaluated during 12 days (7 time points). For Guineo, 77 fruits among 11 hands were evaluated during 24 days (9 time points). For Gros Michel, 93 fruits among 19 hands were evaluated during 14 days (5 time points).

### Physical, chemical and physiological characterisation of the fruit during ripening

In a second experiment, more comprehensive analyses were conducted using a smaller number of fruits and the same storage conditions. Based on the ripening kinetics determined in the first experiment, the following storage days were evaluated: 0, 1, 2, 4, 5 days for Guineo, 0, 2, 4, 6, 8 days for Gros Michel and 0, 2, 4, 6, 8, 10 days for Dominico Harton (day 0 was the harvest day). On average, seven fruits per time point were evaluated.

### Chemical parameters

#### Dry matter content and total soluble solids (TSS)

Dry matter content was determined for the pulp and peel of each variety, and at each time point, while TSS was evaluated only in the pulp, following the protocols reported by Dadzie & Orchard (1997).

#### Total starch content

Total starch content was determined according to Holm *et al*. (1986) with some modifications. Total carbohydrates were determined in lyophilised pulp, using enzymatic hydrolysis (α‐amylase and amyloglucosidase) followed by GOD‐POD reaction and colorimetry at 510 nm. Free glucose was determined using sulphuric acid, amyloglucosidase and GOD‐POD. Total starch was calculated as follows:

TS=C×162/180‐FG

Where, TS: Total starch (% w/w, db). C: Total carbohydrates (% w/w, db). 162/180: Factor to convert C from free glucose, as determined in the aliquot, to anhydroglucose, as occurs in starch. FG: Free glucose (% w/w, db).

Finally total starch (TS) was also reported as percentage (w/w) of the fresh weight of the banana pulp, using dry matter data determined as described in section 2.4.1.

### Colour parameters of the peel

#### Peel colour

Fruit peel colour was measured at different time points during ripening using a portable colour reader (CR‐410, Konica Minolta, Japan). Results were expressed according to the CIELAB system with D65 illuminant, and 10˚ viewing angle, which includes three parameters: L* from 0 (black) to 100 (diffuse white), a* from green (negative values) to red (positive values) and b* from blue (negative values) to yellow (positive values). Preliminary tests indicated that peel colour changed at different rates along the length of the fruit during the ripening process. Therefore, peel colour was reported from the central section of the fruit, taken as representative of the fruit as a whole. Measurements were done in triplicates.

#### Chlorophyll and total carotene content

Peel samples were taken from the central area of the fruits, then ground in liquid nitrogen, extracted for 1 h with 20 mL of 80% acetone (v/v) under dark conditions and refrigeration and then centrifuged at 4538 × g for 10 min. The supernatant was used to determine the chlorophyll and carotenes content spectrophotometrically according to the method of Lichtenthaler (1987). Results were expressed in μg.g^−1^ on a fresh sample weight basis.

### Production of ethylene and respiration rate

Fruits were selected at each time point, weighed and stored in a sealed glass container kept at 23 °C. Before gas sampling, the varieties were equilibrated between 1 and 2 hours. A sample of the headspace gas was collected and injected in a gas chromatographer (Shimadzu^®^ GC‐2014 #13070, Japan), to analyse ethylene and carbon dioxide simultaneously. The system was equipped with a series of stainless steel packed columns operated at 97 °C. The carrier gas was nitrogen (N_2_). Ethylene was measured directly with a flame ionisation detector (FID, 250 °C), while carbon dioxide was converted into methane with a methaniser (380 °C), then measured with the FID. Gas concentrations were calculated using calibration curves and GS‐Solution software (Shimadzu ^®^). Ethylene (C_2_H_4_) production and respiration rates (CO_2_) were expressed in µL kg^−1^ h^−1^ and mg kg^−1^ h^−1^, respectively, in fresh weight basis.

### Statistical analysis

The results of physicochemical and physiological characterisations were reported as the mean of duplicate analyses. Higher number of replicates (constrained by sample availability) was used for those tests with higher inherent variability. Comparison of means was performed by one‐way analysis of variance (ANOVA) followed by Tukey's multiple comparison tests. Correlations between variables were analysed using Pearson's correlation test. Multivariate analysis of variance (MANOVA) was used to compare the different fruit varieties considering all the physicochemical and physiological attributes measured. Linear discriminant analysis (LDA) was used to find differences between fruit varieties based on linear combination of the studied variables (discriminant functions) and the determination of the specific weight of each variable in fruit differentiation. The results obtained were validated using classification functions (Alvin, 2002). Principal component analysis (PCA) was used to determine which variables were responsible for the largest variance in the data. The statistical analyses were done with JMP software (SAS Institute Inc., Version 13 2.1).

## Results and discussion

### Determination of ripening time using colour parameters and total soluble solids (TSS)

**Figure 1:**
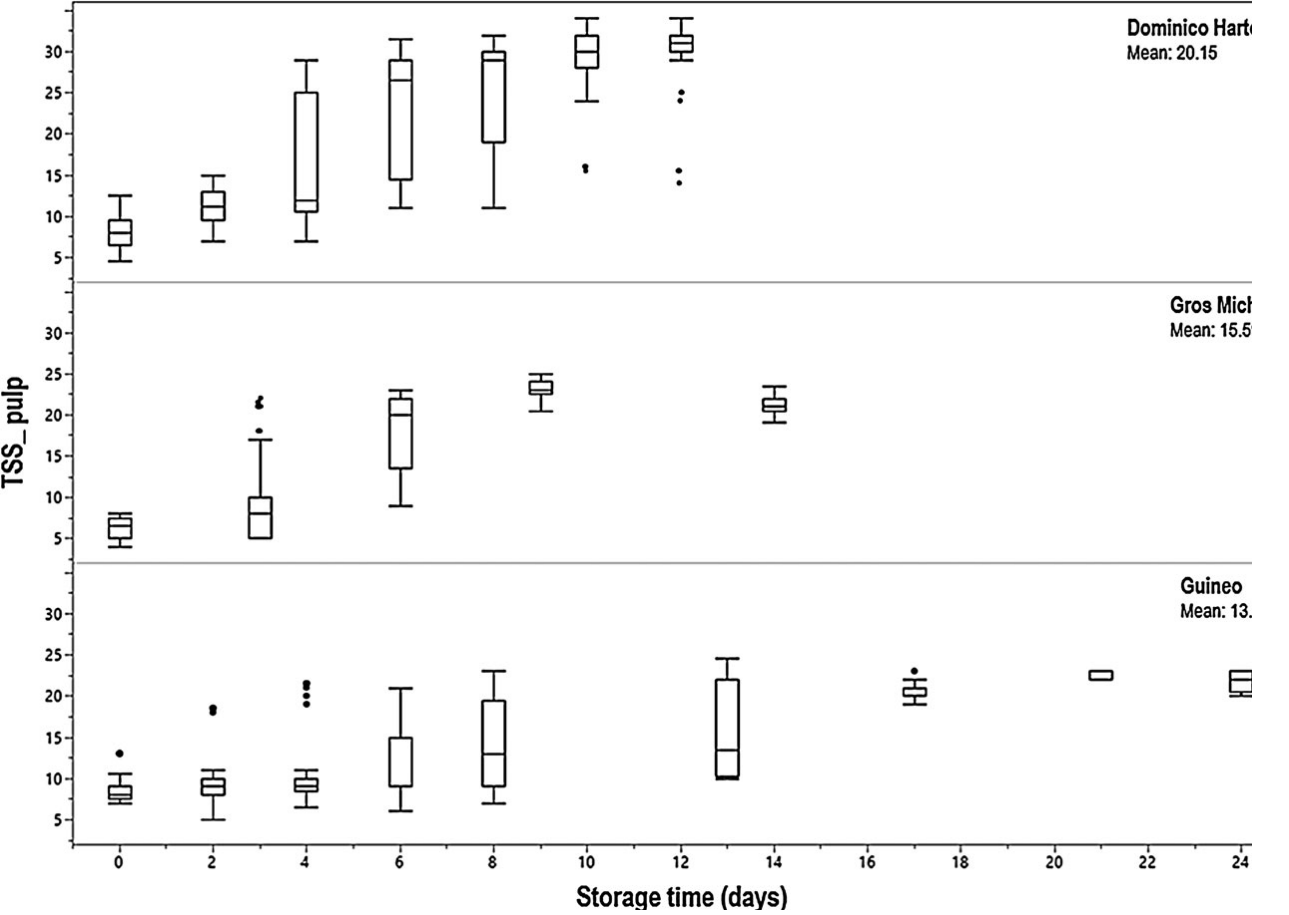
Evolution of total soluble solids (TSS) in banana pulp during post‐harvest storage. Box plots represent the distribution by quartiles of the data. Outliers are shown as individual points and were identified as data outside 1.5 times the interquartile range (1.5×IQR). [Colour figure can be viewed at wileyonlinelibrary.com]

In the first experiment, a total of 314 fruits from the three varieties were evaluated at various storage time points. Between day 0 and day 12 to 14 of storage, luminosity of the peel decreased with the presence of brown patches, and the hue shifted from green‐yellow to red‐yellow (Table 1). The Dominico Harton variety presented the highest luminosity (L*) and colour parameter values, as well as the highest TSS over the whole period of storage. TSS increased over storage time for every variety. Dominico Harton reached maximum TSS on the last day of reported storage (12 days). Gros Michel reached maximum TSS at 9 days of storage, to plateau until the last day of storage (14 days). Some fruit from the Guineo variety reached maximum TSS as early as day 4 of storage, even though the ripening process continued up to 24 days (Fig. 1). Using the data of the three varieties together, a Pearson correlation analysis identified two positive correlations between the colour parameter a* of the peel and TSS in pulp (0.87, *P* < 0.0001), and between colour parameters L* and b* of the peel (0.86, *P* < 0.0001). Non‐destructive measurements of the colour of the peel may therefore be used to predict TSS and the degree of ripening of the fruits, as suggested by Ward & Nussinovitch (1996). Measuring L*, a* and b* colour parameters can also potentially replace the subjective colour charts used in Colombia, which are derived from the one proposed by von Loesecke (1950) for Gros Michel. Colour charts, however, have the advantage of evaluating the fruit as a whole, whereas L*a*b* measurements are more localised on the fruit surface, which can lead to higher data variability when fruit ripening is not homogeneous.

**Table 1:** Comparison of colour parameters of peel and total soluble solids (TSS) of fruit pulp of Guineo, Gros Michel and Dominico Harton varieties between day 0 and day 12–14 of storage

Variety	L^*^‐peel	a^*^‐peel	b^*^‐peel	TSS‐pulp (⁰Brix)
Guineo				
Day 0	31.5 ± 10.3	−17.9 ± 3.2	27.3 ± 4.5	8.8 ± 1.6
Day 13	18.8 ± 6.5	−0.8 ± 8.8	17.6 ± 9	15.8 ± 5.8
Gros Michel				
Day 0	51.2 ± 10.0	−29.3 ± 2.5	50.7 ± 7.7	6.3 ± 1.3
Day 14	29.6 ± 14.5	14.4 ± 6.6	44.5 ± 25.3	21.2 ± 0.9
Dominico Harton				
Day 0	60.5 ± 9.3	−31.4 ± 2.9	63.2 ± 10.5	8.0 ± 1.9
Day 12	29.2 ± 25.4	13.8 ± 11.9	43.4 ± 42.7	30.0 ± 4.1

Data presented are the means ± standard deviation.

In the second experiment, the detailed changes during ripening were studied with a smaller number of fruits.

### Changes in physical characteristics of the fruit during ripening

The fruit of the Dominico Harton variety was characterised by their significantly larger size and weight, compared to the fruits of Gros Michel (intermediate size) and Guineo (small). Statistically significant morphological changes during ripening were reductions in fruit weight, central diameter and peel thickness (Table S1), while the pulp/peel ratio increased, as was also observed by other authors (Ngalani *et al*., 1998; Newilah *et al*., 2009). These changes are explained mainly by water loss from the peel (transpiration), and transfer of water from peel to pulp as starch is converted to sugars that increase osmotic pressure in the pulp (Von Loesecke, 1950; Fernandes *et al*., 1979). The variety and storage time had a significant effect on the fruit weight, central diameter, pulp/peel ratio, peel (%) and peel thickness. Additionally, the interaction of Variety × Storage time (V × ST) was significant for fruit weight (*P* = 0.0017), pulp/peel ratio (*P* = 0.0018) and percentage of peel (*P* = 0.0038), indicating that the evolution of these parameters during ripening depended on the variety of *Musaceae*. On the other hand, the interaction of Variety × Storage time (V × ST) was not significant for fruit length, central diameter and peel thickness, indicating that these parameters evolved in similar fashion during ripening, whatever the variety.

### Changes in chemical parameters during ripening

#### Dry matter content and total soluble solids

The dry matter content of the pulp and peel decreased with storage time for all the evaluated varieties. Dry matter of the pulp of Gros Michel and Guineo was lower than Dominico Harton during the whole storage period (Table 2). TSS increased during fruit ripening for all varieties, in particular in the case of Dominico Harton and Gros Michel varieties. This change is related to the hydrolysis of starch to soluble sugars such as sucrose, glucose and fructose (Marriott *et al*., 1981). In the case of Gros Michel, TSS increased until the sixth day of storage and then decreased at the end of ripening, which can be explained by the conversion of sugars to alcohol (Dadzie & Orchard, 1997). These results were comparable to observations on another dessert banana (Cavendish variety), which presented a sigmoidal evolution of TSS (Wills *et al*., 1984). Variety (V), storage time (ST) and their interaction (VxST) had statistically significant effects on dry matter content in the pulp and the peel (*P* < 0.05, Table 2). However, only ST had a statistically significant effect on TSS, for all varieties (Table 2).

**Table 2:** Chemical characteristics of fruits from Dominico Harton, Gros Michel and Guineo for different storage times

Variety	ST (days)	Chemical parameters			Starch (%, wb)
		Pulp dry matter (%, wb)	Peel dry matter (%, wb)	TSS‐pulp (⁰Brix)	
Dominico Harton	0	40.2 ± 0.8	16.8 ± 0.2	10.2 ± 1.2	36.4 ± 0.1
	2	40.0 ± 0.2	14.9 ± 0.6	10.0 ± 1.3	35.8 ± 0.0
	4	39.9 ± 0.5	15.0 ± 0.3	12.2 ± 3.1	28.8 ± 0.1
	6	37.6 ± 0.4	14.3 ± 0.3	16.2 ± 1.5	21.3 ± 0.8
	8	37.3 ± 0.7	14.1 ± 0.2	25.2 ± 2.1	12.7 ± 0.2
	10	34.8 ± 0.3	13.1 ± 0.2	32.2 ± 1.7	3.4 ± 0.3
Gros Michel	0	23.5 ± 0.4	10.7 ± 0.5	5.0 ± 0.4	14.8 ± 1.0
	2	23.3 ± 0.8	10.6 ± 0.6	11.9 ± 4.9	5.9 ± 0.3
	4	21.6 ± 0.5	10.5 ± 0.3	19.6 ± 2.2	0.3 ± 0.2
	6	21.3 ± 0.3	10.3 ± 0.4	22.7 ± 0.4	0.1 ± .0.0
	8	20.4 ± 0.4	9.8 ± 0.1	20.6 ± 1.4	0.0 ± 0.0
Guineo	0	30.3 ± 1.1	15.8 ± 0.2	6.8 ± 1.3	19.7 ± 0.1
	1	29.1 ± 1.3	15.4 ± 0.7	9.8 ± 1.8	18.4 ± 0.0
	2	26.3 ± 0.4	14.0 ± 0.5	8.4 ± 0.6	13.4 ± 0.1
	4	26.3 ± 0.8	13.9 ± 0.3	13.8 ± 2.3	5.3 ± .0.0
	5	23.3 ± 1.0	13.8 ± 0.6	19.8 ± 2.0	1.0 ± 0.2
Average		29.7	13.3	15.3	14.5
ST. dev.		7.5	2.2	7.5	12.3
CV (%)		25.1	16.8	49.0	84.6
V		<0.0001	<0.0001	0.5782	<0.0001
ST		<0.0001	<0.0001	<0.0001	<0.0001
VxST		<0.0001	0.0006	0.6269	<0.0001

Data presented are the means ± standard deviation (St. dev.). The statistical significance of Variety (V), Storage time (ST) and Variety‐by‐storage time interactions (VxST) effects on each parameter is presented at the end of the table (*P* < 0.05 indicates a significant effect on the parameter considered).

CV, coefficient of variance; TSS, total soluble solids; wb, weight basis; wt, weight.

#### Total starch content

The three varieties had significantly different (*P* < 0.05) initial starch contents, in the following order: Dominico Hartón, Guineo and Gros Michel (respectively, 36.4% ± 0.1%; 19.7% ± 0.1%; and 14.8% ± 1.0% wb). Starch content decreased during ripening, with different starch hydrolysis rates (Table 2). Starch content in Gros Michel decreased faster, with more than 60% of the initial content hydrolysed by the second day, followed by Guineo and Dominico Hartón. At the end of storage, starch concentrations were 3.40, 1.00 and 0.02 % (wb) for Dominico Hartón, Guineo and Gros Michel, respectively, with significant differences (*P* < 0.0001) between Gros Michel and the other varieties. The degradation of the starch was complete in the Gros Michel variety, whereas Guineo and Dominico Hartón varieties retained small amounts of starch at the end of the ripening period. Several authors have reported similar results in bananas, plantains (triploids) and hybrids (tetraploids) (Marriott *et al*., 1981; Sakyi‐Dawson *et al*., 2008). Several enzymes are associated with starch hydrolysis during ripening, such as amylases, glucosidase, phosphorylase, sucrose synthase and invertase (Cordenunsi & Lajolo, 1995). Xiao *et al*. (2018) identified 38 genes that encode proteins related to the degradation of starch in bananas and found that 27 of these candidate genes were significantly induced during the ripening of banana fruit, with enzyme activities regulated at both transcriptional and translational levels. Variety (V), storage time (ST) and their interaction (VxST) had a statistically significant effect on starch content (Table 2), which we interpreted as follows: (i) Varieties had different levels of starch content, with Dominico Harton presenting approximately twice as much starch as the other varieties at Day 0; (ii) the extent of starch hydrolysis during ripening was significant; and (iii) the progression of starch hydrolysis differed during ripening depending on the variety, with a lower hydrolysis rate observed in plantains (Dominico Harton).

### Changes in the colour of the peel during ripening

#### Peel colour

The fruit colour evolved from green to yellow during storage for the three varieties, as reflected by increases in a* and b* colour parameters (Table 3), with statistically significant effects (*P* < 0.01) of variety and storage time. On the first day of storage, each variety presented different colour parameters, with average values of luminosity (L*) 49.5, 39.9 and 30.9 for Dominico Harton, Gros Michel and Guineo, respectively (Table 3). L* decreased for the Dominico Harton variety over the first days of storage until day 4, to increase until day 8, and decrease again until day 10. This trend was very similar for the Gros Michel variety, reaching its maximum luminosity at day 6. On the other hand, the luminosity of the Guineo variety steadily decreased over the 5 days of storage. The colour parameter b* was always positive (yellow hues) for all the varieties and followed a trend similar to L*, with gradual increase followed by a decrease at the end of the storage (Table 3). This decrease was related to the appearance of brown patches, a physiological phenomenon that takes place in the last stages of fruit ripening, and the transition of peel colour from yellow towards red and, finally, brown or black (Ketsa, 2000). The colour parameter a* was lower for Dominico Harton, reflecting its intense green colour in the early ripening stages, as observed by Kajuna *et al*. (1998). For the studied varieties, the transition of a* from negative values to positive values took place at different storage days (Table 3).

**Table 3:** Changes in the colour parameters (L*, a*, b*) and the chlorophyll and carotenoid content of peel with storage time (ST)

Variety		ST (days)	L*	a*	b*	Total chlorophylls (µg g^−1^)	Total carotenoids (µg g^−1^)	(a + b)/(x + c)
Dominico Harton	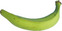	0	49.5 ± 5.1	−29.1 ± 3.6	51.3 ± 8.0	89.7 ± 0.7	28.2 ± 5.1	3.2
	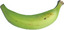	2	46.1 ± 6.9	−27.7 ± 2.6	51.1 ± 7.8	35.5 ± 2.2	19.8 ± 2.3	1.8
	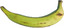	4	45.0 ± 5.7	−23.1 ± 4.2	59.5 ± 9.3	26.6 ± 3.9	22.0 ± 7.5	1.3
	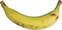	6	57.1 ± 10.2	−8.7 ± 3.3	72.5 ± 12.6	22.0 ± 2.1	28.0 ± 6.7	0.8
	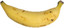	8	76.0 ± 7.8	11.2 ± 4.8	117.1 ± 7.6	12.0 ± 2.9	28.3 ± 3.8	0.4
	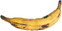	10	36.7 ± 13.0	23.7 ± 3.1	61.0 ± 24.8	8.7 ± 1.2	26.4 ± 1.2	0.3
Guineo	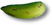	0	30.9 ± 9.2	−17.5 ± 2.8	27.4 ± 4.3	100.7 ± 8.8	28.2 ± 2.0	3.6
	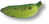	1	30.1 ± 12.0	−15.3 ± 5.1	30.7 ± 14.4	95.8 ± 13.9	28.2 ± 2.9	3.4
	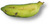	2	24.1 ± 14.8	−12.6 ± 3.9	28.9 ± 26.0	59.7 ± 6.3	26.0 ± 3.4	2.3
	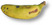	4	19.7 ± 8.7	−8.1 ± 4.5	17.2 ± 10.4	60.6 ± 2.3	20.2 ± 0.5	3.0
	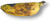	5	4.9 ± 1.7	1.8 ± 0.8	2.0 ± 2.9	36.7 ± 9.3	42.2 ± 9.4	0.9
Gros Michel	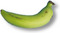	0	39.9 ± 6.5	−26.0 ± 2.7	40.9 ± 6.9	126.5 ± 17.6	36.9 ± 12.7	3.7
	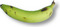	2	35.4 ± 7.1	−22.9 ± 1.9	42.2 ± 6.2	63.9 ± 7.7	23.2 ± 1.2	2.8
	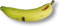	4	45.0 ± 10.9	−12.0 ± 4.3	62.1 ± 16.5	29.1 ± 2.5	31.2 ± 1.8	0.9
	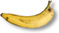	6	56.4 ± 9.5	13.1 ± 2.6	85.4 ± 14.8	11.5 ± 1.2	28.0 ± 2.6	0.4
	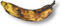	8	13.2 ± 3.6	13.1 ± 2.5	13.2 ± 6.3	7.2 ± 1.4	20.8 ± 4.8	0.4

Statistical significance of the sources of variation (probability> F) from ANOVA						
Variety (V)	<0.0001	<0.0001	<0.0001	0.1012	0.1195	0.2716
Storage time (ST)	<0.0001	<0.0001	0.3958	<0.0001	0.7629	<0.0001
VxST	**<0.0001**	**<0.0001**	**<0.0001**	**0.0003**	**0.0359**	**0.029**

Data presented are the means ± standard deviation (St. dev.). The statistical significance of Variety (V), Storage time (ST) and Variety‐by‐storage time interactions (VxST) effects on each parameter is presented at the end of the table (*P* < 0.05 indicates a significant effect on the parameter considered). The parameter (a + b)/(x + c) is an indicator of senescence based on the relation between the concentrations of chlorophyll and carotenoids (a, chlorophyll a; b, chlorophyll b; x, xanthophyll; c, carotenes).

The different rates of colour change in the three varieties are related to variations in pigments synthesis such as carotenoids, and degradation of chlorophyll (Ammawath *et al*., 2001). In turn, pigment synthesis and degradation are controlled by the levels of expression or activity of the relevant enzymes, such as pheophorbide A Oxygenase in the case of chlorophyll (Yang *et al*., 2009). The genes related with chlorophyll degradation (MaSGR, MaNYC and MaPaO) are sensitive to mild thermal stress (30 °C) (Du *et al*., 2014), so that bananas may develop a brighter yellow appearance when ripened at temperatures below 30 °C, with optimum between 18 and 24 °C. At higher ripening temperatures, the fruit may display a green‐ripe colour unacceptable in the market. Optimum temperature for chlorophyll‐degrading enzymes activity may also depend on variety, since at the storage temperature studied, the Guineo variety did not develop a fully yellow peel, indicating retention of high levels of chlorophyll (Blackbourn *et al*., 1990; Yang *et al*., 2009), in contrast to Dominico Harton.

For the colour parameters L* and a*, all sources of variation (V, ST, VxST) presented significant *F*‐tests; however, the storage time did not show a significant effect (*P‐value* = 0.3958) on the colour parameter b* (Table 3).

#### Chlorophyll and carotenes content in peel

Chlorophyll content in the peel of the three varieties decreased during storage (Table 3). Chlorophyll was highest in the Gros Michel variety at the beginning of storage (126.5 µg g^1^), followed by Guineo and Dominico Harton (100.7 and 89.7 μg g^1^, respectively). During storage, the chlorophyll degraded 94, 90 and 64% for Gros Michel, Dominico Harton and Guineo, respectively. The concentration of carotenoids did not follow a clear trend, but tended to decrease towards the end of the storage period (Table 3).

The ratio between chlorophyll a and chlorophyll b, and total carotenoids (xanthophyll (x) + carotenes (c)), (a + b)/(x + c), is a metric to measure the level of green colour in plants, and can be used as indicator of senescence. Common values for this ratio range between 4.2 and 5 for plants under sun exposure. For the Musaceae varieties in this study, this ratio was low, with initial values between 3.2 and 3.7, respectively, decreasing to 0.3 to 0.9 at the end of storage (Table 3).

Significant differences in the evolution of chlorophyll a and b and total carotenoids were found for different varieties and storage times. Pearson's interaction analysis indicated a link between total chlorophyll and colour parameter a* (*r* = −0.69, *P* = 0.032). Another, less significant correlation was found between total chlorophyll and carotenes (*r* = 0.49, *P* = 0.0553). The storage time only had a significant effect on the total chlorophyll content, while the effects of VxST were significant for the total content of chlorophyll and carotenoids (Table 3).

### Control of ripening: Production of ethylene and respiration rate during ripening

**Figure 2:**
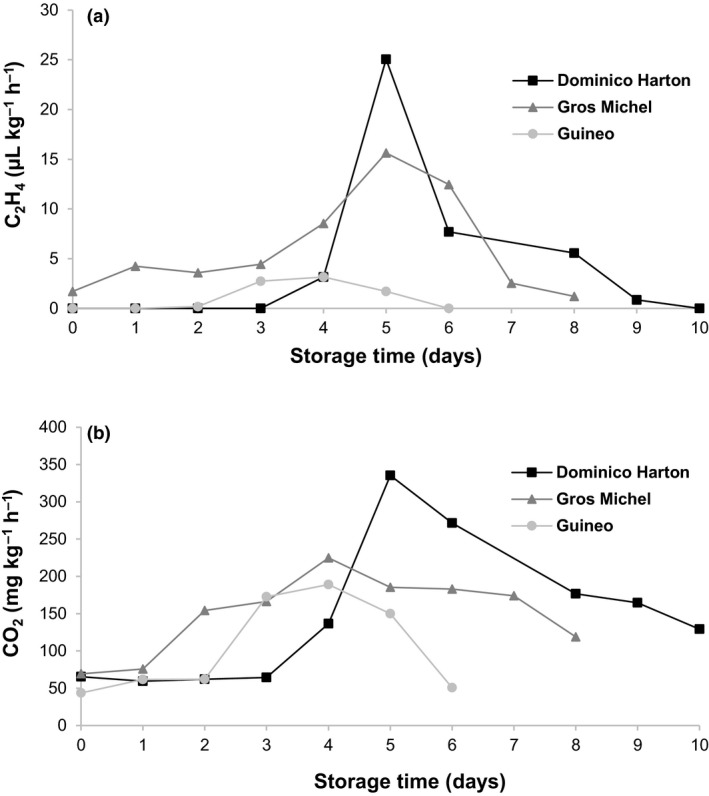
Production rate of ethylene (a) and CO_2_ (b) during storage for three varieties of Musaceae. [Colour figure can be viewed at wileyonlinelibrary.com]

Dominico Harton and Guineo varieties did not present pre‐climacteric detectable ethylene levels, (Fig. 2a), in contrast to Gros Michel with ethylene production of 1.7 µL kg^−1^ h^−1^. For Dominico Harton, ethylene production started on day 3 of storage, with a sharp production of ethylene on day 5 (25.1 µL kg^−1^ h^−1^) marking the beginning of the climacteric phase (Liu *et al*., 1999). After the fifth day of storage, ethylene rapidly decreased back to undetectable levels at day 10. For Guineo, ethylene production started on day 2 of storage and reached maximum production on day 4, with 3.2 µL kg^−1^ h^−1^. After the peak, production steadily decreased until undetectable levels at day 6. For Gros Michel, ethylene production increased steadily over the first days of storage until the maximum on day 5 (15.6 µL kg^−1^ h^−1^). After that, a sharp decrease brought ethylene to almost undetectable levels by day 8.

Respiration rate continuously increased after day 1, 2 and 3 of storage for Gros Michel, Guineo and Dominico Harton varieties, respectively (Fig. 2b). At the beginning of storage, fruit were in pre‐climacteric state with low basal metabolism and respiration rate (43.6‐69.3 mg CO_2_ kg^−1^ h^−1^). When the climacteric phase started, respiration rate increased as well as ethylene production (Fig. 2a), leading to the climacteric autocatalytic peak. The maximum respiration rates were 335.4, 224.7 and 189.0 mg kg^−1^ h^−1^ for Dominico Harton, Gros Michel and Guineo, respectively (Fig. 2b). After the peak, respiration rate decreased back to initial values for the Guineo variety, but remained at higher levels compared to the initial values for the other two varieties until the end of storage.

The physiological changes observed during ripening are triggered during the climacteric phase (Hiwasa *et al*., 2003; Inaba *et al*., 2007). Kader (2002) classified bananas as a high ethylene producer fruit due to the intense metabolic activity inducing ripening. However, in this study, the sharp ethylene and respiration peaks were observed only for the Dominico Harton variety and to a lower extent for Gros Michel (Fig. 2a). Strategies to extend shelf‐life of bananas and plantains are based on the inhibition or control of ethylene production (López‐Gómez *et al*., 1997), in order to reduce or delay the physiological, biochemical and molecular changes that lead to fruit senescence. Some varieties, such as Guineo in the present study, may not produce large amounts of ethylene during their ripening and may be less responsive to ethylene‐inhibition treatments. For such varieties, research on alternative strategies to extend shelf‐life may be beneficial. Monitoring the respiration rate and intensity is also an option to detect some of the physical and biochemical changes during ripening, such as starch hydrolysis into sugars and CO_2_ (Palmer, 1971), and degradation of cell walls. The respiratory intensity and climacteric peak of Musaceae are influenced by storage conditions, age of the fruit and variety, and in turn influence the shelf‐life of the product (Kader, 2002). Controlling respiration through hormonal treatments, such as 1‐methylcyclopropene, applied before harvest or at the beginning of post‐harvest storage, is therefore promising options for controlling shelf‐life (Kader, 2002).

### Statistical analysis

Multivariate analysis of variance (MANOVA) for physicochemical properties showed highly significant differences (*P* < 0.0001) between the Dominico Harton, Guineo and Gros Michel varieties (Table 4). These results indicate that varieties evaluated are different based on variation in physicochemical properties. A linear discriminant analysis was performed to determine the most relevant physicochemical parameters contributing to the differentiation among varieties. Furthermore, the differences among the three varieties (Table 5) were explained by two discriminant functions (DFs). Central diameter, TSS of the pulp, colour parameter a* and total starch contributed most in discriminating the three varieties and accounted for most of the expected variations in physicochemical properties. The classification matrix for the three groups (Table 6) showed that 100% of the cases were correctly classified to their respective groups, confirming that the physicochemical parameters can be used to differentiate among varieties.

**Table 4:** Multivariate analysis of variance (MANOVA) tests performed on the three *Musaceae* varieties in the study

Effect	Test	Value	*F*	Prob> F
Group	Wilk's Lambda	0.01	63.84	<0.0001
	Pillai's trace	1.79	61.44	<0.0001
	Hotelling's Trace	18.34	66.43	<0.0001
	Roy's largest root	12.11	89.44	<0.0001

**Table 5:** Coefficients of the discriminant functions (DF) for the three fruit varieties studied (Dominico Harton, Gros Michel and Guineo)

Parameter	DF 1	DF 2
Fruit weight	0.73	0.10
Length inner arch	0.06	−0.86
Length outer arch	0.35	0.28
Central diameter	−0.67	0.76
Peel (%wt.)	0.00	0.00
Pulp/peel (%wt/%wt)	−0.14	−0.22
Peel thickness	−0.04	0.74
TSS pulp	1.49	0.77
L^*^ peel	−0.05	−0.51
a^*^ peel	0.21	1.78
b^*^ peel	0.15	−0.09
Total starch	1.52	1.17

**Table 6:** Classification table for the discriminant analysis of the three varieties studied

Group	Error (%)	Predicted values		
		Gros Michel	Dominico Harton	Guineo
Gros Michel	0.0	35	0	0
Dominico Harton	0.0	0	36	0
Guineo	0.0	0	0	39

**Figure 3:**
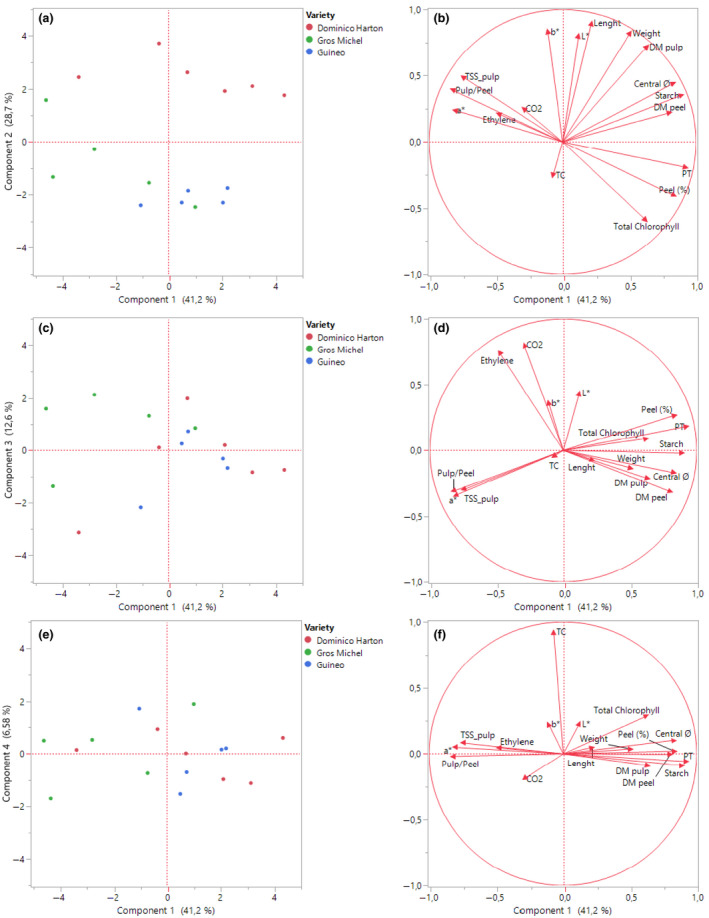
Principal component analysis (PCA) of the physicochemical and physiological variables characterised for three *Musaceae* varieties. Bi‐plots represent PC2 vs. PC1 (a), PC3 vs. PC1 (c) and PC4 vs. PC1 (e). Vector representation of variables along principal components shows the contribution of the studied variables in the definition of principal components: PC2 vs. PC1 (b), PC3 vs. PC1 (d) and PC4 vs. PC1 (f). TC, total carotenes; DM, dry matter; Ø, diameter; PT; peel thickness; TSS, total soluble solids; L*, a* and b*, colour parameters.

Principal component analysis of the physicochemical and physiological characteristics reduced the dimensionality of the data from 17 variables to 4 principal components capturing 89.08% of the total data variability (Fig. 3). The bi‐plot of the second principal component (PC2) vs. the first (PC1) (Fig. 3a) showed two clusters scattered along PC2: Dominico Harton for positive values of PC2, and Gros Michel and Guineo for negative values. The clustering of Gros Michel and Guineo varieties together reflects their similar biophysical characteristics, in particular the variables related to fruit size (fruit weight and length) and colour parameter (L* and b*), which were the main contributors to PC2 (Fig. 3b). Variables contributing mainly to PC1 were diameter in the central section, total soluble solids in pulp, peel colour parameter a*, peel thickness, peel (%wt.), dry matter content in peel and total starch content in pulp. Principal components PC3 and PC4 did not distinguish further clusters of samples (Fig. 3d and f). Respiration parameters (ethylene and CO_2_ production) and carotene content were the main variables contributing, respectively, to PC3 and PC4.

## Conclusions

This study focused on the physicochemical and physiological differences between *Musaceae* varieties during ripening. Simple, objective and reproducible tests were identified to characterise the ripening process of bananas and plantains. In particular, non‐destructive measurements such as central diameter and colour parameter a* were well correlated with, and can therefore predict, quality indicators of the ripening stage such as TSS and total starch of the pulp. This study contributes to address some important gaps in research on banana post‐harvest handling, including the development of novel tests to evaluate more comprehensively the changes during ripening, and the characterisation of the ripening not only of two major varieties Dominico Harton and Gros Michel, but also of Guineo, an economically important variety for domestic markets that is less studied than export varieties. The post‐harvest storage conditions in this study were at ambient temperature, to reflect local market conditions and constraints in Colombia and generate practical information for better post‐harvest handling processes. Indeed, most banana producers are small farmers who do not have access to storage and transport facilities with controlled atmosphere.

Further research may focus on interactions between genotype and storage conditions: Both variety and storage conditions influence ripening and lead to a wide range of ripening behaviours, which are not fully elucidated. In particular, the ripening of banana in bunches can be different from bananas stored as individual fruits or as hands; and the behaviour of bunches of bananas can vary from progressive ripening starting at one end over several days, to sudden ripening of the whole bunch within a few hours. The analytical approaches developed in this study can be applied to better understand the kinetics of banana ripening, by characterising the behaviour of various *Musaceae*. Further studies to expand the data sets available on ripening of bananas will help developing reliable prediction models of the ripening stages and fruit quality, applicable to a large number of genotypes.

## Conflict of interest

The authors declare that they have no conflict of interest.

## Author contribution

**Jhon Larry Moreno:** Data curation (lead); Formal analysis (lead); Investigation (lead); Methodology (lead); Writing‐original draft (lead); Writing‐review & editing (equal). **Thierry Tran:** Data curation (equal); Formal analysis (equal); Funding acquisition (supporting); Supervision (equal); Writing‐original draft (equal); Writing‐review & editing (equal). **Borja Cantero:** Writing‐original draft (equal); Writing‐review & editing (supporting). **Karina Lopez:** Project administration (equal); Supervision (equal). **Luis Augusto Becerra:** Project administration (equal); Resources (equal); Supervision (equal). **Dominique Dufour:** Funding acquisition (equal); Project administration (equal); Resources (equal); Supervision (equal).

## Ethical guidelines

Ethics approval was not required for this research.

### Peer Review

The peer review history for this article is available at https://publons.com/publon/10.1111/ijfs.14851.

## Supporting information

Table S1. Physical characteristics of fruits from Dominico Harton, Gros Michel, and Guineo varieties for different storage times

## Data Availability

Data available on request from the authors.
