# Supplementary material for: Physicochemical and physiological changes during the ripening of Banana (Musaceae) fruit grown in Colombia
Source: Int J Food Sci Technol. 2020 Nov 3;56(3):1171–83. doi: 10.1111/ijfs.14851 (PMC7984252; doi:10.1111/ijfs.14851)
Supplement: Supplementary file 1 — Table S1. Physical characteristics of fruits from Dominico Harton, Gros Michel, and Guineo varieties for different storage times [file IJFS-56-1171-s001.docx]

**Supplementary Material**

**Physicochemical and Physiological Characterization of the Maturation of Musaceae Fruit Grown in Colombia**

Jhon Larry Moreno ^1,3*^, Thierry Tran ^1,5^, Borja Cantero ^2^, Karina López ^4^, Luis Augusto Becerra Lopez Lavalle ^1^, Dominique Dufour ^5^

**^1^** The Alliance of Bioversity International and the International Center for Tropical Agriculture (CIAT), CGIAR Research Program on Roots Tubers and Bananas (RTB), Apartado Aéreo 6713, Cali, Colombia

**^2^** Robert Frederick Smith School of Chemical and Biomolecular Engineering, Cornell University, Ithaca, NY 14850, USA

**^3^** Facultad de Ingeniería y Administración**,** Universidad Nacional de Colombia. Carrera 32 # 12-00, Palmira, Valle del Cauca, Colombia

**^4^** Facultad de Ciencias Agropecuarias**,** Universidad Nacional de Colombia. Carrera 32 # 12-00, Palmira, Valle del Cauca, Colombia

**^5^** Qualisud, University of Montpellier, CIRAD, Montpellier SupAgro, University of Avignon, University of La Réunion, 73 rue JF Breton, 34398 Montpellier, France.

Supplementary Table S1: Physical characteristics of fruits from Dominico Harton, Gros Michel, and Guineo varieties for different storage times

| **Variety** | **ST (days)** | **Physical parameters** | | | | | |  |
| --- | --- | --- | --- | --- | --- | --- | --- | --- |
|  |  | **Fruit weight (g)** | **Length (cm)** | **Central Diameter (cm)** | **Pulp/Peel (wt/wt)** | **Peel (%, wt)** | **Peel thickness (mm)** |  |
| Dominico Harton | 0 | 374.1 ± 47.2 | 30.0 ± 2.4 | 5.2 ± 0.1 | 1.4 ± 0.2 | 41.6 ± 2.8 | 4.1 ± 0.2 |  |
|  | 2 | 354.9 ± 15.9 | 29.6 ± 1.2 | 5.0 ± 0.1 | 1.6 ± 0.1 | 37.9 ± 1.3 | 3.9 ± 0.1 |  |
|  | 4 | 306.2 ± 41.4 | 26.5 ± 2.6 | 4.8 ± 0.2 | 1.7 ± 0.1 | 37.2 ± 2.0 | 3.9 ± 0.4 |  |
|  | 6 | 318.9 ± 37.2 | 27.2 ± 1.8 | 4.7 ± 0.2 | 1.6 ± 0.4 | 39.4 ± 5.8 | 3.3 ± 0.5 |  |
|  | 8 | 300.9 ± 46.8 | 27.1 ± 2.1 | 4.7 ± 0.2 | 2.0 ± 0.6 | 34.4 ± 5.9 | 3.2 ± 0.2 |  |
|  | 10 | 256.9 ± 37.6 | 26.3 ± 1.8 | 4.2 ± 0.2 | 3.4 ± 0.6 | 23.1 ± 2.8 | 1.6 ± 0.1 |  |
| Gros Michel | 0 | 160.2 ± 18.8 | 21.2 ± 0.7 | 4.1 ± 0.2 | 1.4 ± 0.1 | 42.3 ± 1.4 | 3.4 ± 0.3 |  |
|  | 2 | 147.9 ± 19.7 | 20.2 ± 1.6 | 3.9 ± 0.1 | 1.7 ± 0.2 | 37.7 ± 2.3 | 3.3 ± 0.2 |  |
|  | 4 | 148.2 ± 21.2 | 20.5 ± 1.1 | 3.9 ± 0.2 | 2.0 ± 0.1 | 33.3 ± 1.4 | 2.4 ± 0.1 |  |
|  | 6 | 166.2 ± 9.4 | 22.9 ± 0.7 | 3.9 ± 0.1 | 3.1 ± 0.2 | 24.6 ± 1.0 | 2.0 ± 0.2 |  |
|  | 8 | 116.1 ± 11.4 | 20.1 ± 0.4 | 3.5 ± 0.1 | 2.7 ± 0.3 | 26.9 ± 2.2 | 1.5 ± 0.4 |  |
| Guineo | 0 | 173.3 ± 21.2 | 17.9 ± 1.5 | 4.7 ± 0.3 | 1.5 ± 0.1 | 40.0 ± 1.9 | 4.0 ± 0.1 |  |
|  | 1 | 141.4 ± 24.8 | 16.2 ± 1.7 | 4.4 ± 0.3 | 1.3 ± 0.2 | 44.7 ± 3.8 | 4.2 ± 0.5 |  |
|  | 2 | 152.8 ± 21.6 | 17.3 ± 1.3 | 4.4 ± 0.3 | 1.5 ± 0.1 | 40.8 ± 1.9 | 4.0 ± 0.2 |  |
|  | 4 | 133.5 ± 32.0 | 16.0 ± 1.7 | 4.3 ± 0.4 | 1.3 ± 0.2 | 43.8 ± 3.7 | 4.0 ± 0.5 |  |
|  | 5 | 164.5 ± 19.7 | 17.9 ± 1.9 | 4.4 ± 0.2 | 1.8 ± 0.3 | 35.6 ± 3.7 | 3.0 ± 0.6 |  |
| Average |  | 205.8 | 21.8 | 4.4 | 1.9 | 36.5 | 3.2 |  |
| ST. dev. |  | 87.1 | 4.8 | 0.5 | 0.7 | 7.0 | 0.9 |  |
| CV (%) |  | 42.3 | 22.2 | 11.3 | 36.1 | 19.3 | 28.7 |  |
| **V** | | <0.0001 | <0.0001 | <0.0001 | <0.0001 | <0.0001 | <0.0001 |  |
| **ST** |  | <0.0001 | 0.0115 | <0.0001 | <0.0001 | <0.0001 | <0.0001 |  |
| **VxST** |  | 0.0017 | 0.1054 | 0.0651 | 0.0018 | 0.0038 | 0.1993 |  |

Data presented are the means ± standard deviation (St. dev.). CV, coefficient of variance; wt, weight; wb, weight basis; TSS, total soluble solids. The statistical significance of Variety (V), Storage time (ST) and Variety-by-storage time interactions (VxST) effects on each parameter is presented at the end of the table (p < 0.05 indicates a significant effect on the parameter considered).
